# Association of Neck Circumference With Cardiometabolic Risk Factors and Diseases in the German National Cohort

**DOI:** 10.1210/jendso/bvaf163

**Published:** 2025-10-22

**Authors:** Eike A Strathmann, Ilka Ratjen, Klara Willrodt, Janna Enderle, Sabrina Schlesinger, Beate Fischer, Katharina S Weber, Cara Övermöhle, Karin H Greiser, Anja M Sedlmeier, Margit Heier, Anna Köttgen, Kathrin Günther, Matthias Nauck, Wolfgang Lieb

**Affiliations:** Institute of Epidemiology and Biobank popgen, Christian-Albrechts-University, University Hospital Schleswig-Holstein Campus Kiel, Kiel 24105, Germany; Clinic for Internal Medicine II, Hematology and Oncology, University Hospital Schleswig-Holstein Campus Kiel, Kiel 24105, Germany; Institute of Epidemiology and Biobank popgen, Christian-Albrechts-University, University Hospital Schleswig-Holstein Campus Kiel, Kiel 24105, Germany; Institute for Human Nutrition and Food Science, Christian-Albrechts-University, Kiel 24105, Germany; Institute for Biometrics and Epidemiology, German Centre for Diabetes, Heinrich-Heine-University, Düsseldorf 40225, Germany; German Center for Diabetes Research, Partner Düsseldorf, München-Neuherberg 85764, Germany; Institute for Epidemiology and Preventive Medicine, University of Regensburg, Regensburg 93053, Germany; Institute of Epidemiology and Biobank popgen, Christian-Albrechts-University, University Hospital Schleswig-Holstein Campus Kiel, Kiel 24105, Germany; Institute of Epidemiology and Biobank popgen, Christian-Albrechts-University, University Hospital Schleswig-Holstein Campus Kiel, Kiel 24105, Germany; Division of Cancer Epidemiology, German Cancer Research Center (DKFZ), Heidelberg 69120, Germany; Institute for Epidemiology and Preventive Medicine, University of Regensburg, Regensburg 93053, Germany; Center for Translational Oncology, University Hospital Regensburg, Regensburg 93053, Germany; Institute of Epidemiology, Helmholtz Zentrum München–German Research Center for Environmental Health (GmbH), Neuhersberg 85764, Germany; KORA Study Centre, University Hospital of Augsburg, Augsburg 86153, Germany; Institute of Genetic Epidemiology, Department of Data Driven Medicine, Faculty of Medicine and Medical Center-University of Freiburg, Freiburg 79106, Germany; Department of Epidemiological Methods and Etiological Research, Leibniz Institute for Prevention Research and Epidemiology–BIPS, Bremen 28359, Germany; Institute for Clinical Chemistry and Laboratory Medicine, University Medicine Greifswald, Greifswald 17475, Germany; DZHK (German Centre for Cardiovascular Research), Partner Site Greifswald, University Medicine, Greifswald 17475, Germany; Institute of Epidemiology and Biobank popgen, Christian-Albrechts-University, University Hospital Schleswig-Holstein Campus Kiel, Kiel 24105, Germany

**Keywords:** cardiovascular disease, metabolic disease, diabetes, German National Cohort, NAKO, cardiometabolic risk factors

## Abstract

**Context:**

Neck circumference (NC) was proposed as promising marker to assess body fat distribution and cardiometabolic risk.

**Objective:**

We aimed to assess associations of NC with anthropometric traits, cardiometabolic risk markers, and self-reported cardiometabolic diseases.

**Methods:**

NC was measured in a subsample (5865 participants) of the German National Cohort (NAKO Gesundheitsstudie, NAKO), study region Kiel. Linear and logistic regression models were applied to assess associations of NC with anthropometric and cardiometabolic risk markers and self-reported cardiometabolic diseases, including diabetes, heart failure, gout, and a composite end point “clinical CVD” (cardiovascular disease; combining history of angina pectoris, stroke, myocardial infarction, and peripheral artery disease). Models were adjusted for sex and age, CV risk factors (systolic blood pressure, diabetes, low-density lipoprotein [LDL] cholesterol, use of lipid-lowering and antihypertensive medication, smoking status), and body mass index (BMI).

**Results:**

Mean NC values (±SD) were 39.5 ± 3.0 in men and 33.6 ± 2.7 cm in women. NC was positively associated with anthropometric traits, visceral adipose tissue (cm) (β = 1.45 [95% CI, 0.88-2.02]), systolic (β = .37 [0.19-0.56]) and diastolic (β = .17 [0.05-0.29]) blood pressure, glycated hemoglobin A_1c_ (β = .02 [0.01-0.02]), nonfasting glucose (β = .57 [0.31-0.83]), and inversely associated with high-density lipoprotein cholesterol (β = −.73 [−0.91; −0.54]). Furthermore, NC showed associations with diabetes (odds ratio [OR] = 1.08 [1.02-1.15]), heart failure (OR = 1.12 [1.02-1.23]), and gout (OR = 1.09 [1.01-1.17]). Association with “clinical CVD” did not remain statistically significant after BMI adjustment.

**Conclusion:**

NC was associated with several cardiometabolic risk factors, including glycemic and lipid traits and self-reported cardiometabolic diseases. These observations suggest that NC may be a useful surrogate marker for cardiometabolic risk.

Current estimates indicate that more than 603.7 million adults are obese, and therefore, have an increased risk of developing disease conditions such as diabetes, cardiovascular diseases (CVDs), and certain forms of cancer [1]. Abdominal fat can be subdivided into subcutaneous (SAT) and visceral adipose tissue (VAT), which differ in their metabolic activity and confer differential risks for the aforementioned disease conditions [2]. In particular, VAT, which surrounds the inner organs, secretes adipocytokines and other vasoactive substances that confer increased CV risk [3, 4]. Common measures of overweight and obesity are body mass index (BMI), as well as waist circumference (WC), a marker of abdominal fat, which are associated with obesity-related health risk [5]. Both measures are relatively simple to obtain and therefore applicable in clinical practice and large epidemiological studies [6]. Furthermore, body fat can quickly and accurately be assessed via bioelectric impedance analysis (BIA). In combination with BMI, body fat can further be used to calculate two additional measures that are both associated with diabetes and prediabetes, the fat mass index (FMI) and the fat-free mass index (FFMI) [7]. However, all of these measures lack the ability to mirror differences in the distribution of abdominal body fat compartments (SAT and VAT), which can been measured accurately only using imaging technologies, such as ultrasound, magnetic resonance imaging, and computed tomography, that are expensive and time-consuming to perform [8]. Neck circumference (NC), a promising marker of abdominal fat, is highly correlated both with BMI and WC [9]. More important, it was found to be highly correlated with VAT and associated risk factors for CVD [9]. Furthermore, longitudinal and cross-sectional studies from North and South America, Europe, and Asia indicate that NC is associated with increased risk of type 2 diabetes and increased blood pressure [9-12]. Additionally, NC was found to be associated with increased serum urate levels and hyperuricemia, suggesting associations between NC and risk for gout [13].

The aim of our study was to determine the distribution of NC in the general population and to examine its association with anthropometric traits, cardiometabolic risk markers, and prevalent cardiometabolic diseases as well as gout in a large community-based sample, the Kiel regional subsample of the German National Cohort (NAKO Gesundheitsstudie).

## Materials and Methods

### Study Design and Sample

**Figure 1.**
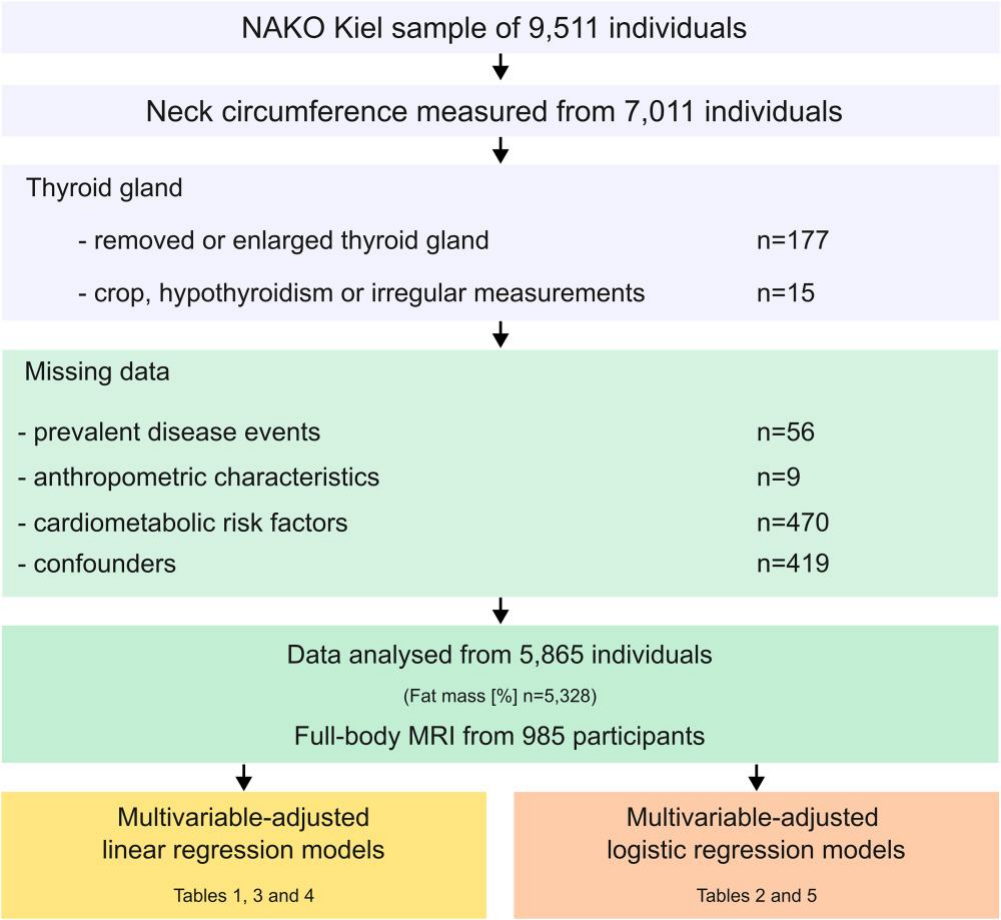
Schematic diagram of study participants and exclusion criteria.

The present analyses are based on a subset (n = 9511) of participants in NAKO, Germany's largest population-based cohort study with more than 205 415 participants, aged 19 to 74 years, recruited in 18 different study centers across the country [14]. Participants were extensively characterized by a variety of medical examinations and tests, standardized interviews and questionnaires, as described in detail elsewhere [14]. Additionally, a broad spectrum of biosamples was collected from each participant [14]. NC was measured in 7011 participants of the NAKO Kiel sample. Data from individuals whose thyroid gland was completely or partially removed or enlarged (n = 177), as well as individuals with a goiter, hypothyroidism, or with irregularities during the measurement of NC (n = 15) were excluded. Furthermore, individuals with missing data on prevalent disease events (n = 56), anthropometric characteristics (n = 9), cardiometabolic risk markers (n = 470) as well as missing potential confounders (n = 419) were excluded, resulting in an analytical sample of 5865 participants. FM measurements from BIA and ultrasound-based measurements of VAT and SAT were available from 5328 and 985 participants, respectively (Fig. 1).

### Phenotypic Characterization of the Participants

Participants visited the NAKO study center at the University Hospital Schleswig-Holstein in Kiel, where a standardized clinical and physical examination, including anthropometric measurements, and a comprehensive standardized computer-assisted face-to-face interview were conducted by trained personnel, collecting information on sex, age, and use of medications during the past 7 days, among others. Furthermore, participants completed self-administered touch-screen questionnaires [14].

NC was measured horizontally subapical of the thyroid cartilage, using the inelastic tape measure SECA 201 with an accuracy of 0.1 cm. To analyze interrater and intrarater reliability, 2 examiners measured NC in duplicates in a small group of 9 participants. For the interrater reliability, intraclass correlation coefficients (ICCs) of 0.899 and ICCs of 0.96 were obtained for the 2 examiners, respectively. Reproducibility between the 2 examiners was then tested by intraclass correlation in a 2-way random-effects model. Our analysis shows an excellent agreement between the average NC measurements of examiner 1 and examiner 2 with an ICC of 0.904 (*P* = .002). In agreement with the published literature, NC measurements were highly reproducible [15-17]. WC was measured at the midpoint between the lower edge of the thoracic cage and the upper edge of the ilium, using the same tape. In 97.4% of participants (n = 5712), weight and height were measured at the study center. In 2.6% of cases (n = 153), data on these characteristics were self-reported. Weight (accuracy 0.1 kg) was assessed using the medical Body Composition Analyzer 515 (mBCA 515, SECA). Body height (cm) was measured using the SECA Stadiometer 274 with an accuracy of 0.1 cm. Body FM [%] was determined by BIA at a frequency of 50 kHz [18]. The Omron 705IT (HEM-759-E, Omron Healthcare GmbH) was used to record systolic and diastolic blood pressures (mm Hg). After a 5-minute rest in a sitting position, 2 consecutive measurements spaced 2 minutes apart were performed, and the second blood pressure measurement was used for the present statistical analyses. VAT and SAT were measured in duplicates by ultrasound using the Philips iE33 (Philips GmbH) with 1 to 5 MHz ultrasonic transducers. The study participants were placed in a supine position, while the ultrasonic probe was placed on the midpoint between the lowest costal reach and the tip of the iliac crest. To determine SAT (cm), the distance between the surface of the skin and the linea alba was measured. VAT (cm) was defined as the distance between the linea alba and the front of the lumbar vertebra [18]. The day of the visit in the study center blood was drawn for the measurement of several cardiovascular risk markers, including nonfasting glucose (mg/dL), glycated hemoglobin A_1c_ (HbA_1c_) (%), total cholesterol (mg/dL), high-density lipoprotein (HDL) cholesterol and low-density lipoprotein (LDL) cholesterol (mg/dL), and serum urate (mg/dL). Serum lipoproteins were directly measured by enzyme assay photometry using a Dimension VISTA 1500 (Siemens Healthineers) platform. Additionally, participants were asked to report prevalent cardiometabolic disease conditions (from a predefined list of physician-diagnosed medical conditions, including diabetes, gout, heart failure, angina pectoris, myocardial infarction, peripheral artery disease, and apoplectic stroke) [19].

### Derived Variables

BMI was calculated as weight in kilograms divided by the square of height in meters (kg/m^2^). FMI was calculated as FM divided by squared height (kg/m^2^). FFMI was calculated as BMI subtracted by FMI (kg/m^2^).

### Statistical Analysis

All statistical analyses were performed using R (version 4.2.3) and RStudio (2023.03.1 Build 4446). Categorical measurements were reported as absolute numbers and percentages (n [%]). Normality of data distribution for all variables was assessed by visual inspection using quantile-quantile plots. Normally distributed continuous variables were reported as mean and SD, not normally distributed variables as median, Q1 to Q3. To compare the frequency of categorical variables between groups, chi-square tests were conducted. All figures were generated using the R package “ggplot2.”

#### Model and confounder selection

Literature-based confounder selection was performed and candidates were grouped into base characteristics (age and sex), classical cardiometabolic risk factors (systolic blood pressure, antihypertensive medication, LDL cholesterol, lipid-lowering medication [yes; no], [self-reported] diabetes [yes; no], smoking [smoker; former smoker]), socioeconomic characteristics (alcohol consumption [g/day], years of education [≤10 years; 11-13 years; 14-17 years; ≥18 years]), and BMI [20]. All linear and logistic regression models were adjusted for sex and age (model 1), additionally for classical cardiometabolic risk factors (model 2), and additionally for BMI (model 3), except for the anthropometric traits and the measures of body fat distribution, which were intercorrelated with BMI. To ensure the robustness of the results, a sensitivity analysis was performed in a subsample (n = 5406 participants), in which alcohol consumption and years of education were considered as additional confounders.

#### Association of neck circumference with anthropometric traits and body fat distribution

First, we assessed the association of various anthropometric measures (BMI, height, weight, and WC) and measures of body fat distribution (SAT, VAT, fat mass, FMI, FFMI; as exposure variables) with NC (as outcome variable) in multivariable-adjusted linear regression models. To allow for comparisons of the calculated effect estimates (β coefficients), the values of the exposure and confounder variables were Z-transformed (mean of 0, SD of 1) and fit to the models as exposure variables (each exposure separately). Hence, β coefficients and 95% CIs reflect the change in NC (in cm) per 1-SD increment of the exposure variable. Model assumptions (such as linearity and normality of residuals) were checked visually for all models using quantile-quantile plots.

#### Association of neck circumference with cardiometabolic risk markers and serum urate

Next, we analyzed if NC (as exposure variable) was associated with known cardiometabolic risk markers (systolic and diastolic blood pressures, HbA_1c_, nonfasting glucose, total cholesterol, HDL cholesterol, and LDL cholesterol and with serum urate; each variable considered as separate outcome) using linear regression models. Model assumptions (such as linearity and normality of residuals) were checked visually for all models using quantile-quantile plots.

#### Association of neck circumference with clinical cardiovascular events diabetes and gout

The associations of NC (as exposure) with binary outcome variables (self-reported CV and metabolic disease events and gout) were examined using multivariable-adjusted logistic regression analysis. Self-reported cardiometabolic disease events included angina pectoris, myocardial infarction, peripheral artery disease, and apoplectic stroke. In most cases, these disease entities are ischemic and thus have a common pathophysiological basis. Due to the small number of individual events, myocardial infarction, angina pectoris, apoplectic stroke and peripheral artery disease were combined into one composite end point “clinical CVD.” Diabetes, heart failure, and gout were used as separate outcome variables (each outcome modeled individually).

### Ethical Statement

This study was approved by the ethical review board of the Medical Faculty of Kiel University (AZ 106/13). All participants gave written informed consent.

## Results

### Sample Characteristics

**Figure 2.**
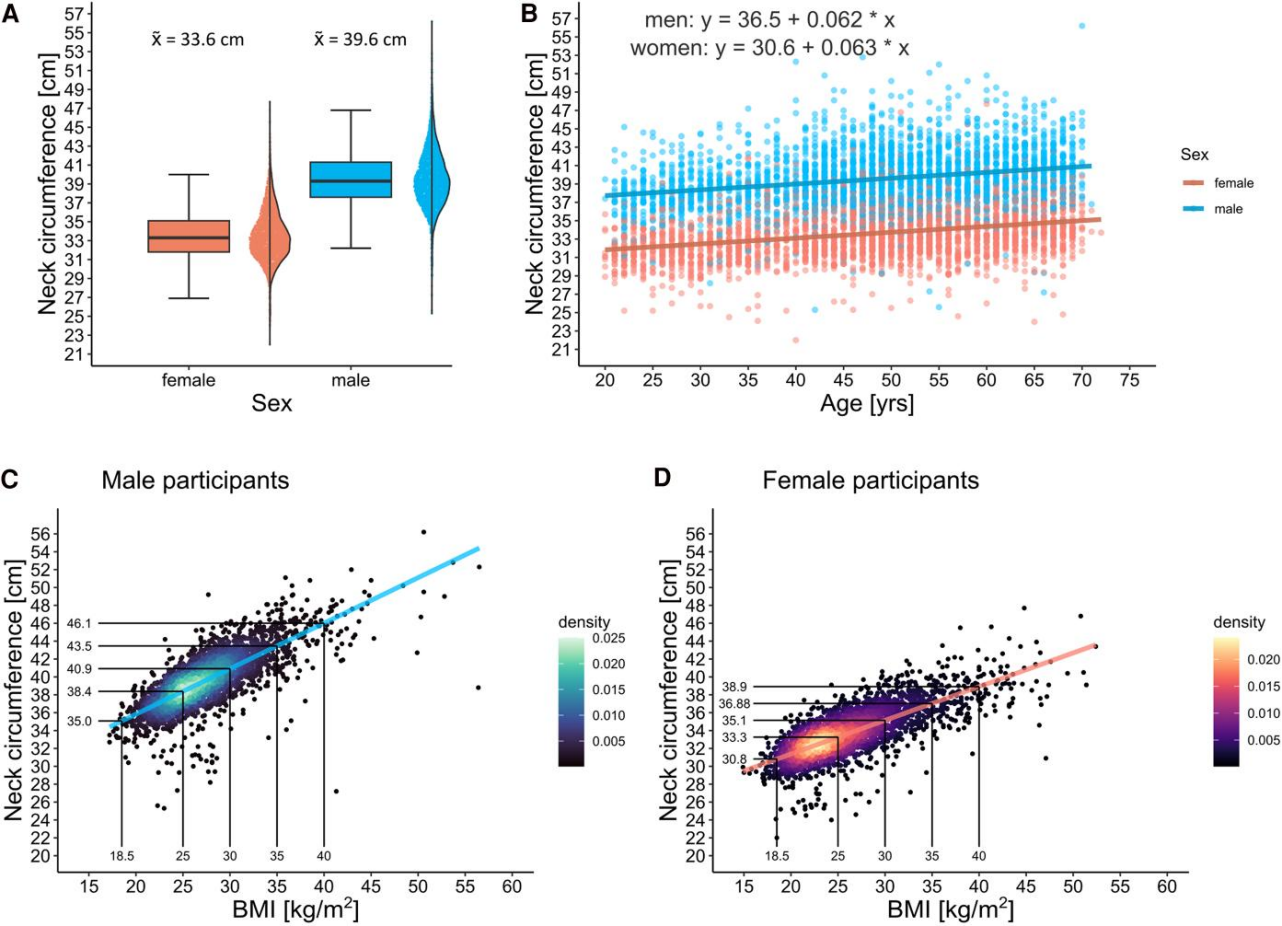
Correlation of neck circumference (NC) with age, sex, and body mass index (BMI). A, Box plots and half-violin plots of 5865 NC measurements in male (n = 2992) and female (n = 2873) study participants. Mean NCs of 39.5 ± 3.0 cm in men and 33.6 ± 2.7 cm were measured. B, Scatterplot of NC (cm) on the y-axis and age (years) on the x-axis. In both sexes, the NC increased with age in a linear manner by 0.06 cm per year of age. C and D, Scatterplot of NC (cm) on the y-axis and BMI on the x-axis. A 2-dimensional kernel density estimation indicates the density of data points at a given section of the plot. There is a linear correlation between BMI and NC for C, men and D, women, respectively.

Our analytical dataset included 5865 participants (49% women) with a median age of 49 years (Table 1). The measured mean NCs were 39.6 ± 3 cm in men and 33.6 ± 2.7 cm in women (Fig. 2A). NC increased with age in a linear fashion by 0.06 cm per year of age (Fig. 2B). About one-fifth of participants were obese (BMI ≥ 30; n = 1176; 20.1%). In both sexes, NC was highly correlated with BMI (Fig. 2C and 2D). Women had a 10% higher amount of body fat, while men were more often obese (BMI: 26.7 [24.3-29.5]) compared to women (BMI: 24.7 [95% CI, 22.1-28.5]), and had an increased VAT/SAT ratio of 3.5 (a.u.) (compared to 2.4 (a.u.) in women). Besides that, men and women showed similar levels of glycemic traits (nonfasting glucose and HbA_1c_), while there were sex-specific differences in lipid traits, with higher LDL cholesterol levels, but lower HDL cholesterol levels in men (see Table 1). Self-reported history of clinical CVD events (myocardial infarction, angina pectoris, apoplectic stroke, and peripheral artery disease) was relatively uncommon (Table 2). About 5% (n = 297) of the participants reported one of the diseases of the primary composite end point “clinical CVD.” This end point was statistically significantly more common in men (7.2%; n = 216) than in women (2.8%; n = 81; *P* ≤ .001). The most common (metabolic) disease in our sample was diabetes (n = 271; 4.6%; see Table 2) with similar prevalences in both sexes. Gout (n = 199; 3.4%) and heart failure (n = 124; 2.1%) were relatively uncommon (see Table 2).

**Table 1.** Baseline characteristics of study participants

Base characteristics	Men	Women
Sex	2992 (51.0%)	2873 (49.0%)
Age, y	49 (41-59)	49 (38-58)
**Anthropometric traits**		
Neck circumference, cm	39.5 ± 3.0	33.6 ± 2.7
BMI	26.7 (24.3-29.5)	24.6 (22.1-28.5)
Height, cm	180.2 ± 7.1	167.1 ± 6.7
Weight, kg	86.7 (78.0-97.2)	68.8 (61.5-80.0)
SAT, cm*^a^*	1.9 ± 0.7	2.1 ± 1.0
VAT, cm*^a^*	6.6 ± 2.3	5.0 ± 2.1
Fat mass, %*^b^*	25.5 ± 7.0	35.5 ± 7.9
Fat mass index, kg/m^2^*^b^*	6.8 ± 3.0	8.6 ± 4.0
Fat-free mass index, kg/m^2^*^b^*	19.9 ± 1.9	16.0 ± 1.7
**Medication**		
Hypertension	819 (27.4%)	578 (20.1%)
Antihypertensive medication		
Yes	600 (20.1%)	447 (15.6%)
No	2392 (79.9%)	2426 (84.4%)
Hyperlipidemia	603 (20.2%)	531 (18.5%)
Lipid-lowering medication		
Yes	231 (7.7%)	133 (4.6%)
No	2761 (92.3%)	2740 (95.4%)
**Cardiometabolic risk factors**		
Blood pressure		
Systolic, mm Hg	131.6 ± 14.3	122.5 ± 15.1
Diastolic, mm Hg	81.5 ± 9.6	77.5 ± 9.3
Smoking status		
Never	1236 (41.3%)	1442 (50.2%)
Previous smoker	1119 (37.4%)	899 (31.3%)
Current smoker	637 (21.3%)	532 (18.5%)
HbA_1c_, %	5.4 (5.3-5.6)	5.4 (5.2-5.6)
Glucose, mg/dL	93.7 (88.3-104.5)	90.1 (82.9- 97.3)
Total cholesterol, mg/dL	201.3 ± 40.5	207.4 ± 41.2
LDL cholesterol, mg/dL	126.6 ± 33.8	122.2 ± 34.7
HDL cholesterol, mg/dL	51.9 ± 12.9	67.5 ± 16.6
Serum urate, mg/dL	5.5 ± 1.1	4.0 ± 1.0

Categorical measurements are reported as absolute numbers and percentages (n, (%)). Normally distributed continuous variables are reported as mean ± SD ($\bar{x}$ ± SD), or otherwise as median (Q1-Q3).

Abbreviations: BMI, body mass index; HbA_1c_, glycated hemoglobin A_1c_; HDL, high-density lipoprotein; LDL, low-density lipoprotein; SAT, subcutaneous adipose tissue; VAT, visceral adipose tissue.

^a^Ultrasound measurements available in a subpopulation of (n_total_ = 985, n_men_ = 472, n_women_ = 513) participants.

^b^Fat mass was measured by bioelectric impedance analysis in a subpopulation of (n_total_ = 5328, n_men_ = 2731, n_women_ = 2597) participants.

**Table 2.** Prevalent self-reported cardiovascular and metabolic diseases in the overall sample and in men and women

Self-reported cardiometabolic diseases	Total	Men	Women
n	5865	2992	2873
Composite primary end point (CVD)*^a^*			
Yes	297 (5.1%)	216 (7.2%)	81 (2.8%)
No	5531 (94.3%)	2760 (92.2%)	2771 (96.4%)
**Other prevalent cardiometabolic diseases**			
Diabetes			
Yes	271 (4.6%)	154 (5.1%)	117 (4.1%)
No	5594 (95.4%)	2838 (94.9%)	2756 (95.9%)
Gout			
Yes	199 (3.4%)	150 (5%)	49 (1.7%)
No	5666 (96.6%)	2842 (95%)	2824 (98.3%)
Heart failure			
Yes	124 (2.1%)	74 (2.5%)	50 (1.7%)
No	5741 (97.9%)	2918 (97.5%)	2823 (98.3%)
**Individual CVD end points**			
Angina pectoris			
Yes	125 (2.1%)	102 (3.4%)	23 (0.8%)
No	5724 (97.6%)	2880 (96.3%)	2844 (99%)
Unknown	16 (0.3%)	10 (0.3%)	6 (0.2%)
Myocardial infarction			
Yes	87 (1.5%)	72 (2.4%)	15 (0.5%)
No	5768 (98.3%)	2914 (97.4%)	2854 (99.3%)
Unknown	10 (0.2%)	6 (0.2%)	4 (0.1%)
Peripheral artery disease			
Yes	81 (1.4%)	46 (1.5%)	35 (1.2%)
No	5772 (98.4%)	2940 (98.3%)	2832 (98.6%)
Unknown	12 (0.2%)	6 (0.2%)	6 (0.2%)
Apoplectic stroke			
Yes	75 (1.3%)	54 (1.8%)	21 (0.7%)
No	5779 (98.5%)	2933 (98%)	2846 (99.1%)
Unknown	11 (0.2%)	5 (0.2%)	6 (0.2%)

Measurements were reported as absolute numbers and percentages (n (%)).

Abbreviation: CVD, cardiovascular disease.

^a^Primary composite end point was reached if one or multiple of the following disease events were present: angina pectoris, myocardial infarction, peripheral artery disease, or apoplectic stroke.

### Association of Neck Circumference With Anthropometric Traits and Body Fat Distribution

In multivariable-adjusted linear regression models, NC was positively associated with all anthropometric traits, including BMI, weight, height, and WC, even after multivariable adjustment (model 2; Table 3). Furthermore, there were positive associations of NC with VAT, fat mass, FMI, and FFMI, in age- and sex-adjusted models and in multivariable-adjusted models (model 2; see Table 3). SAT was associated with NC only in age- and sex-adjusted models, but not on further adjustment for CV risk factors (see Table 3).

**Table 3.** Association of neck circumference with various anthropometric traits and measures of body fat distribution

Exposure	Outcome	Model	β (95% CI) per SD	*P*	Adj *r^2^*
Associations of anthropometric traits with NC					
BMI	NC, cm	Model 1	2.05 (2.00 to 2.10)	≤ .001	0.79
		Model 2	1.98 (1.93 to 2.04)	≤ .001	0.79
Height, cm	NC, cm	Model 1	.60 (0.51 to 0.70)	≤ .001	0.57
	NC, cm	Model 2	.66 (0.57 to 0.76)	≤ .001	0.61
Weight, kg	NC, cm	Model 1	2.31 (2.26 to 2.36)	≤ .001	0.8
	NC, cm	Model 2	2.22 (2.17 to 2.28)	≤ .001	0.8
Waist	NC, cm	Model 1	2.39 (2.33 to 2.44)	≤ .001	0.8
circumference, cm	NC, cm	Model 2	2.34 (2.28 to 2.39)	≤ .001	0.8
Associations of NC with measures of body fat distribution					
SAT, cm*^a^*	NC, cm	Model 1	.93 (0.78 to 1.08)	≤ .001	0.62
	NC, cm	Model 2	.43 (−0.18 to 1.04)	.173	0.47
VAT, cm*^a^*	NC, cm	Model 1	1.70 (1.55 to 1.85)	≤ .001	0.71
	NC, cm	Model 2	1.45 (0.88 to 2.02)	≤ .001	0.59
Fat mass, %*^b^*	NC, cm	Model 1	1.99 (1.91 to 2.07)	≤ .001	0.7
	NC, cm	Model 2	1.86 (1.78 to 1.94)	≤ .001	0.71
Fat mass index, kg/m^2*b*^	NC, cm	Model 1	1.95 (1.89 to 2.01)	≤ .001	0.75
	NC, cm	Model 2	1.87 (1.81 to 1.93)	≤ .001	0.76
Fat-free mass index, kg/m^2*b*^	NC, cm	Model 1	2.52 (2.44 to 2.60)	≤ .001	0.75
	NC, cm	Model 2	2.39 (2.31 to 2.47)	≤ .001	0.76

Data are β coefficients from multiple linear regression analyses with 95% CIs. β indicates the change in NC (in cm) per 1 SD of the exposure variable. Model 1 was adjusted for age and sex (men = 0); model 2 was additionally adjusted for cardiometabolic risk factors (systolic blood pressure, antihypertensive medication [yes = 1]; LDL cholesterol; lipid-lowering medication [yes = 1]; diabetes [yes = 1]; smoking [smoker; former smoker]). Adj *r^2^* coefficient of determination adjusted for the number of predictors in the model.

Abbreviations: LDL, low-density lipoprotein; NC, neck circumference; SAT, subcutaneous adipose tissue; VAT, visceral adipose tissue.

^a^Bioimpedance analysis measurements available in a subpopulation of (n_total_ = 985, n_men_ = 472, n_women_ = 513) participants.

^b^Fat mass was measured in a subpopulation of (n_total_ = 5328, n_men_ = 2731, n_women_ = 2597) participants.

Sensitivity analyses, adding alcohol consumption and years of education to model 2, yielded results consistent with the main observations with only minor changes in the effect estimates (Supplementary Table S1) [21]. These sensitivity analyses also showed that BMI was the single most influential confounder (see Supplementary Tables S1 and S2) [21].

### Association of Neck Circumference With Cardiometabolic Risk Markers and Serum Urate

NC displayed consistent and statistically significant associations with several CVD risk factors, including systolic and diastolic blood pressure, HDL and LDL cholesterol, glycemic traits (HbA_1c_ and nonfasting glucose), and serum urate (model 2; Table 4). These associations persisted on multivariable adjustment, except for the association of NC with LDL cholesterol, which was rendered statistically nonsignificant on adjustment for BMI (model 3; see Table 4). There was no association between NC and total cholesterol (model 3; see Table 4). Additional adjustment of model 2 for alcohol consumption and years of education did not alter the results (see Supplementary Table S1) [21].

**Table 4.** Association of neck circumference with various cardiometabolic risk markers

Exposure	Outcome	Model	β (95% CI)	*P*	Adj *r^2^*
Associations of NC with blood pressure					
NC, cm	Systolic blood pressure, mm Hg	Model 1	.78 (0.65 to 0.91)	≤ .001	0.19
		Model 2*^a^*	.62 (0.49 to 0.76)	≤ .001	0.2
		Model 3*^a^*	.37 (0.19 to 0.56)	≤ .001	0.2
NC, cm	Diastolic blood pressure, mm Hg	Model 1	.51 (0.42 to 0.59)	≤ .001	0.11
		Model 2*^a^*	.46 (0.37 to 0.54)	≤ .001	0.54
		Model 3*^a^*	.17 (0.05 to 0.29)	.006	0.54
Associations of NC with cardiovascular risk markers					
NC, cm	HbA_1c_, %	Model 1	.04 (0.04 to 0.05)	≤ .001	0.17
		Model 2	.03 (0.02 to 0.03)	≤ .001	0.41
		Model 3	.02 (0.01 to 0.02)	≤ .001	0.41
NC, cm	Nonfasting glucose, mg/dL	Model 1	1.44 (1.24 to 1.63)	≤ .001	0.11
		Model 2	.84 (0.66 to 1.03)	≤ .001	0.29
		Model 3	.57 (0.31 to 0.83)	≤ .001	0.29
NC, cm	Total cholesterol, mg/dL	Model 1	.33 (−0.03 to 0.69)	.072	0.12
		Model 2	−.65 (−0.83 to −0.48)	≤ .001	0.81
		Model 3	−.03 (−0.28 to 0.21)	.783	0.81
NC, cm	HDL cholesterol, mg/dL	Model 1	−1.76 (−1.89 to −1.63)	≤ .001	0.3
		Model 2	−1.59 (−1.73 to −1.46)	≤ .001	0.32
		Model 3	−.73 (−0.91 to −0.54)	≤ .001	0.34
NC, cm	LDL cholesterol, mg/dL	Model 1	.93 (0.62 to 1.23)	≤ .001	0.1
		Model 2*^b^*	1.22 (0.90 to 1.53)	≤ .001	0.12
		Model 3*^b^*	.15 (−0.29 to 0.58)	.514	0.13
Associations of NC with serum urate					
NC, cm	Serum urate, mg/dL	Model 1	.11 (0.11 to 0.12)	≤ .001	0.41
		Model 2	.10 (0.09 to 0.11)	≤ .001	0.42
		Model 3	.03 (0.02 to 0.05)	≤ .001	0.44

Data are β coefficients from multiple linear regression analyses with 95% CIs. β indicates the change in NC (in cm) per unit of the exposure variable. Model 1 was adjusted for age and sex (men = 0); model 2 was additionally adjusted for cardiometabolic risk factors (systolic blood pressure, antihypertensive medication [yes = 1]; LDL cholesterol; lipid-lowering medication [yes = 1]; diabetes [yes = 1]; smoking [smoker; former smoker]); model 3 was additionally adjusted for BMI. Adj *r^2^* coefficient of determination adjusted for the number of predictors in the model.

Abbreviations: BMI, body mass index; HbA_1c_, glycated hemoglobin A_1c_; HDL, high-density lipoprotein; LDL, low-density lipoprotein; NC, neck circumference.

^a^Not adjusted for systolic blood pressure.

^b^Not adjusted for LDL cholesterol.

### Association of Neck Circumference With Clinical Cardiovascular Disease Events, Diabetes, and Gout

In age- and sex-adjusted models, as well as in models with additional adjustment for cardiometabolic traits, NC was associated with prevalent diabetes, gout, and heart failure. All of these associations were attenuated but remained statistically significant on further adjustment for BMI (model 3; Table 5). Furthermore, NC was associated with a composite end point “clinical CVD” in multivariable-adjusted models (odds ratio [OR] = 1.07 [1.02-1.12]; model 2). However, this association was rendered statistically nonsignificant on further adjustment for BMI (model 3; see Table 5). The analyses of individual CVD end points revealed that only angina pectoris remained associated with NC in fully adjusted models (model 3; Supplementary Table S3) [21]. Apoplectic stroke and peripheral artery disease were not associated with NC (see Supplementary Table S3) [21]. Additional adjustment of model 2 for alcohol consumption and years of education did not alter the results (see Supplementary Table S2) [21].

**Table 5.** Cross-sectional association of neck circumference with clinical cardiovascular disease, diabetes mellitus, heart failure, and gout

Exposure	Outcome	Cases/total	Model	OR (95% CI)	*P*
NC	Composite primary*^a^*	297/5865	Model 1	1.10 (1.05-1.14)	≤ .001
	end point, CVD		Model 2	1.07 (1.02-1.12)	.004
			Model 3	1.05 (0.98-1.12)	.171
NC	Diabetes	271/5865	Model 1	1.27 (1.22-1.33)	≤ .001
			Model 2	1.23 (1.18-1.28)	≤ .001
			Model 3*^b^*	1.08 (1.02-1.15)	.013
NC	Gout	199/5865	Model 1	1.22 (1.17-1.28)	≤ .001
			Model 2	1.18 (1.13-1.24)	≤ .001
			Model 3	1.09 (1.01-1.17)	.019
NC	Heart failure	124/5865	Model 1	1.16 (1.10-1.23)	≤ .001
			Model 2	1.13 (1.06-1.20)	≤ .001
			Model 3	1.12 (1.02-1.23)	.016

Data are ORs with 95% CIs from logistic regression analyses with prevalent disease events (yes = 1) as the dependent variable. Model 1 was adjusted for age and sex (men = 0); model 2 was additionally adjusted for cardiometabolic risk factors (systolic blood pressure; antihypertensive medication [yes = 1]; LDL cholesterol; lipid-lowering medication [yes = 1]; diabetes mellitus [yes = 1]; smoking [smoker; former smoker]); model 3 was additionally adjusted for BMI.

Abbreviations: BMI, body mass index; CVD, cardiovascular disease; LDL, low-density lipoprotein; NC, neck circumference; OR, odds ratio.

^a^Primary composite end point was reached if one or multiple of the following disease events were present: angina pectoris, myocardial infarction, peripheral artery disease, or apoplectic stroke.

^b^Model 3 not adjusted for diabetes.

## Discussion

In a large sample of 5865 participants from the general population, we assessed the association of NC with anthropometric traits, cardiometabolic risk factors, and self-reported cardiometabolic disease events.

### Neck Circumference Is Strongly Associated With Anthropometric Traits

As expected, NC was highly correlated with age and BMI in both sexes. Furthermore, NC was associated with various anthropometric markers, such as weight, body FM, WC, and height, in good agreement with prior studies [22-24]. This nominates NC as a useful marker of general obesity that may offer some unique benefits compared to other anthropometric traits. For example, WC can be influenced by postprandial abdominal distension, respiratory movement, or pregnancy [25-27]. Moreover, measuring WC is becoming more challenging and prone to errors in severely obese individuals because the exact body sites for measurement are difficult to localize underneath the excess abdominal fat tissue [28]. By contrast, NC assessment is not affected by the aforementioned factors and is simple and inexpensive to measure, stable throughout the day, and probably more accepted by conservative individuals and populations [22, 25, 29]. Due to the strong linear association between NC and BMI, it may be useful to define cutoff values for NC to be used for risk prediction similar to the BMI classification system. For example, in our cohort a BMI of 30 (classified as the cutoff for obesity) would correspond to an NC of 40.9 cm in men and 35.1 cm in women (see Fig. 2C and 2D). Similar ideas have been proposed by several research groups: In a Chinese study, an NC of 39 and 35 cm corresponded to a BMI of 30 for men and women, respectively [30]. An Indian study proposed lower values of 35.25 cm in men and 34.25 cm in women [31]. Prospective analyses are warranted to assess potential predictive performance of NC per se and in particular of potential cutoff points for NC.

### Neck Circumference Is Associated With Visceral Abdominal Fat

In our sample, NC was associated with VAT but not with SAT, an observation that is in line with previous reports [3, 4]. For example, in a Chinese general population study, NC was cross-sectionally associated with visceral obesity [32]. Furthermore, in a Spanish cohort of young adults, NC was associated with several anthropometric traits as well as VAT and FMI [33]. Finally, Preis et al [9] observed a significant correlation between greater NC and VAT, but not SAT, even after adjustment for BMI in the Framingham Heart Study. This is of importance since VAT is more metabolically active than SAT and has a strong effect on CVD and diabetes risk [3, 4].

### Neck Circumference Is Associated With Cardiovascular Disease Risk Factors

In line with other studies, NC was associated with several CVD risk factors, including systolic blood pressure, HbA_1c_, nonfasting glucose, HDL cholesterol, and serum urate, even after adjustment for relevant confounders and BMI [24, 25]. Similar observations were reported from the Framingham Heart Study Offspring Cohort and a recent Turkish study of people with type 2 diabetes [9, 34]. In addition, in our sample, NC was associated with total, LDL, and HDL cholesterol in age- and sex-adjusted models, but only the association with HDL cholesterol levels persisted on additional adjustment for BMI. These observations are in line with data from the San Juan Overweight Adults Longitudinal Study, which also reported inverse associations of NC with HDL cholesterol, as well as with a study by Albassam et al [24 , 25] that reported strong correlations of the NC with HDL cholesterol, insulin, and the homeostatic model assessment of insulin resistance (HOMA-IR) at least in overweight and obese individuals. Another study in 305 morbidly obese women (median BMI 44.2), confirmed associations of NC with cardiometabolic risk factors (including HOMA-IR, triglycerides, and HDL cholesterol) [35]. The majority of studies reported positive associations between NC and LDL cholesterol similar to our results [9, 24, 25, 35]. However, a recent Turkish study of 464 adults aged 18 to 65 years with type 2 diabetes mellitus reported an inverse relationship with LDL cholesterol that was statistically significant only in men [34]. In line with recent literature, our study confirms positive associations of NC with systolic and diastolic blood pressure [36-39]. In conclusion, there is solid evidence for consistent associations of NC with blood pressure and lipid and glycemic traits, in particular with HDL cholesterol and HbA_1c_.

### Neck Circumference Is Associated Prevalent Diabetes and Gout

In our sample, in age- and sex-adjusted and in multivariable-adjusted models, NC was associated with prevalent diabetes, heart failure, and gout. However, no clear association of NC with a composite end point “clinical CVD” was found in fully adjusted statistical models. These observations are consistent with analyses in the San Juan Overweight Adults Longitudinal Study cohort [25]. In the Framingham Offspring cohort, NC was associated with incident type 2 diabetes, even after adjustment for BMI, and also led to an improvement in model performance measures [40]. In our sample, a combined end point “clinical CVD” comprising cases with a history of myocardial infarction, apoplectic stroke, peripheral artery disease, or angina pectoris, was associated with NC only in age- and sex-adjusted models, but not after considering established CVD risk factors in the model. Similar results were reported in the Framingham Heart Study, in which NC was not associated with incident CVD in multivariable-adjusted models [9]. Finally, a Chinese study reported positive associations of NC with serum urate levels and hyperuricemia [13]. Our data support this observation, as we observed strong positive associations of NC with serum urate and gout. Together, this indicates that NC could be a useful marker for CV and metabolic risk as well as a predictive marker for diabetes, heart failure, and gout.

### Strengths and Limitations

Strengths of this study include its large population-based design and the comprehensive clinical assessment of anthropometric parameters and cardiometabolic risk factors using well-established standard operating procedures. An important limitation is that clinical events were self-reported from a predefined list of medical conditions that were diagnosed by a physician, according to the participant. In the published literature, the sensitivity, specificity, and positive and negative predictive values for self-reported CVD events varied substantially [41-44]. Importantly, we think that the degree of misclassification in CVD events would not differ by NC and would therefore be nondifferential. In addition to that, the prevalence of cardiometabolic diseases in our study cohort was lower than in other population-based samples in Germany [19]. Furthermore, SAT and VAT measurements were available only for a subcohort of 985 participants.

### Conclusion

NC is an anthropometric measure that is inexpensive and simple to obtain. We observed consistent associations of NC with cardiometabolic risk factors, especially with lipids and glycemic traits, prevalent diabetes, heart failure, and gout. These associations persisted even after adjustment for BMI. Our observations suggest that NC may be a useful surrogate marker for cardiometabolic risk, a premise that warrants further investigations, particularly in longitudinal settings.

## Data Availability

The data are available on reasonable request and can be requested at https://transfer.nako.de/transfer/index.

## Abbreviations

BIA: bioelectric impedance analysis
BMI: body mass index
CVD: cardiovascular disease
FFMI: fat-free mass index
FMI: fat mass index
HOMA-IR: homeostatic model assessment of insulin resistance
HbA_1c_: glycated hemoglobin A_1c_
HDL: high-density lipoprotein
ICC: intraclass correlation coefficient
LDL: low-density lipoprotein
MetS: metabolic syndrome
NAKO: German National Cohort
NC: neck circumference
SAT: subcutaneous adipose tissue
VAT: visceral adipose tissue
WC: waist circumference
